# Supplementary material for: Algebraic graph-assisted bidirectional transformers for molecular property prediction
Source: Nat Commun. 2021 Jun 10;12:3521. doi: 10.1038/s41467-021-23720-w (PMC8192505; doi:10.1038/s41467-021-23720-w)
Supplement: Supplementary file 1 — Supplementary Information [file 41467_2021_23720_MOESM1_ESM.pdf]

Supplementary Information
Algebraic Graph-assisted Bidirectional Transformers for Molecular Property Prediction

Dong Chen[1,2], Kaifu Gao[2], Duc Duy Nguyen[3], Xin Chen[1], Yi Jiang[1], Guo-Wei Wei *[2,4,5] and Feng Pan †[1]

[1] *School of Advanced Materials, Peking University, Shenzhen Graduate School, Shenzhen 518055, China*
[2] *Department of Mathematics, Michigan State University, MI, 48824, USA*
[3] *Department of Mathematics, University of Kentucky, KY 40506, USA*
[4] *Department of Electircal and Computer Engineering, Michigan State University, MI 48824, USA*
[5] *Department of Biochemistry and Molecular Biology, Michigan State University, MI 48824, USA*

This document contains supplementary information about methods and results which were not necessary to include in the central part of the paper but might be of interest to readers. This supplementary material includes the following sections:

Contents

1 Supplementary Note 1 **2**

2 Supplementary Note 2 **4**

3 Supplementary Note 3 **5**
3.1 Input processing . . . 5
3.2 Bidirectional transformer model parametrization . . . 5
3.3 Algebraic graph model parametrization . . . 7
3.4 Feature fusion . . . 9
3.5 Downstream machine learning algorithms . . . 9

4 Supplementary Note 4 **14**

5 Supplementary Note 5 **17**

*Corresponding author: weig@msu.edu
†Corresponding author: panfeng@pkusz.edu.cn

1 Supplementary Note 1

In this work, we use CheMBL [1] as the pre-trained dataset. CheMBL is a chemical database of bioactive molecules with drug-like properties and it is a free database open to the public. [2] The CheMBL26 is the current version of ChEMBL, updated in March 2020. There are over 1.9 million molecules in the CheMBL26 dataset.

Supplementary Table 1: The summary of all datasets.

	Datasets	Task	Total	Train	Validation	Test
**Unlabeled data (pre-train)**	CheMBL	Pre-training	1936342	1926342	10000	-
**Labeled data (fine-tune)**	LD50*[3]	Regression	7413	5931	-	1482
	IGC50[4, 5, 3]	Regression	1792	1434	-	358
	LC50†[3]	Regression	823	659	-	164
	LC50DM†[3]	Regression	353	283	-	70
	Log$P$[6]	Regression	8605	8199	-	406
	FreeSolv[7]	Regression	643	513	65	65
	Lipophilicity[7]	Regression	4200	3360	420	420
	BBBP[7]	Classification	2042	1631	204	204

*The LD50 dataset was originally from https://chem.nlm.nih.gov/chemidplus/ and used in Ref. [3];
†LC50 and LC50DM datasets wereoriginally from http://cfpub.epa.gov/ecotox/ and used in Ref. [3]

For downstream tasks, four toxicity datasets were studied in our work, namely oral rate LD50, 40 h Tetrahymenapyriformis IGC50, 96 h fathead minnow LC50, and 48 h Daphnia Magna LC50DM, the basic information of toxicity datasets are shown in Supplementary Table 1. Among them, LD50 measures the number of chemicals that can kill half of the rats when orally ingested. The LD50 represents the amount of chemicals that can kill half of the rats when orally ingested. It was originally from https://chem.nlm.nih.gov/chemidplus/. IGC50 records the 50% growth inhibitory concentration of Tetrahymena pyriformis organism after 40h.[4, 5] LC50 reports at the concentration of test chemicals in the water in milligrams per liter that cause 50% of fathead minnows to die after 96h. The last one is LC50DM, which represents the concentration of test chemicals in the water in milligrams per liter that cause 50% Daphnia Magna to die after 48h. LC50 and LC50DM were originally from http://cfpub.epa.gov/ecotox/. The unit of toxicity reported in these four data sets is $-\log_{10}$ mol/L. The sizes of these four data sets vary from 353 to 7413, which poses a challenge for a predictive model to achieve consistent accuracy and robustness. For the partition coefficient, the octanol-water partition coefficients, prediction task, the training set contained 8199 molecules and the test set included 406 components. All components in the test set were approved as organic drugs by the Food and Drug Administration (FDA). The log$P$ values, for all training and test sets were compiled by Cheng et al.[6], and all log$P$ values ranged from -4.64 to 8.42 (Supplementary Table 1).

The three datasets Free Solvation (FreeSolv), Lipophilicity, and the Blood–brain barrier penetration (BBBP) are derived from the work of MoleculeNet[7]. ESOL contains 1128 molecules paired with aqueous solubility. This dataset has been used to estimate aqueous solubility directly from molecular structure.[8]. The FreeSolv dataset contains 643 compounds, and the labels include both experimental and calculated hydration free energy of small molecules in water.[9] The unit of the label is kilocalorie per mole (kcal/mol). Lipophilicity is a dataset contains 4200 compounds, which are derived from ChEMBL database[10]. The measured octanol/water distribution coefficient (logD) of the compound was used as the label. In this study, we applied for different random numbers and split the dataset into training, validation, and test datasets 10 times according to the ratio of 80/10/10. The split ratio of the dataset is the same as that used by MoleculeNet[7]. For the task of classification, the Blood-brain barrier penetration (BBBP) dataset is used in this study. BBBP contains 2042 small molecules and original from a study on the modeling and prediction of

the barrier permeability.[11] The binary labels for compound permeability properties are used in this study. Following MoleculeNet[7], scaffold splitting is used to split the BBBP dataset into training, validation, and test set follows the ratio of 80/10/10.

Supplementary Table 2: A total of 51 symbols are used to split SMILES strings

**Index**	0	1	2	3	4	5	6	7	8	9
**Symbol**	c	C	(	)	O	1	2	=	N	@
**Index**	10	11	12	13	14	15	16	17	18	19
**Symbol**	[	]	n	3	H	F	4	-	S	Cl
**Index**	20	21	22	23	24	25	26	27	28	29
**Symbol**	/	s	o	5	+	#	.	\	Br	6
**Index**	30	31	32	33	34	35	36	37	38	39
**Symbol**	P	I	7	Na	%	8	B	9	Si	0
**Index**	40	41	42	43	44	45	46	47	48	49
**Symbol**	Se	K	se	Li	As	Zn	Ca	Mg	Al	Te
**Index**	50									
**Symbol**	te									

Additionally, we statistic the length of SMILES for all molecules. As listed in Supplementary Table 2, a total of 51 symbols are used to split these SMILES strings. The distribution of SMILES string lengths in the CheMBL is shown in Supplementary Figure 1**a**, and the majority of SMILES are within 254 in length. Therefore, in this work, we choose data with SMILES length no greater than 254 to pre-train. The exact number in the training set is 1,926, 342, and 10 thousand SMILES strings were randomly selected as a validating set. The basic information of CheMBL used in pre-training is shown in Supplementary Table 1. The distributions of SMILES string lengths for toxicity and log$P$ datasets are shown in Supplementary Figure 1**b**. Only one SMILES string on the LD50 dataset has a length of more than 254, with a length of 284. Therefore, in the downstream tasks, we truncate these sequences that exceeded the limit length and input only the first 254 symbols. All these datasets are also available at `https://weilab.math.msu.edu/DataLibrary/3D/`.

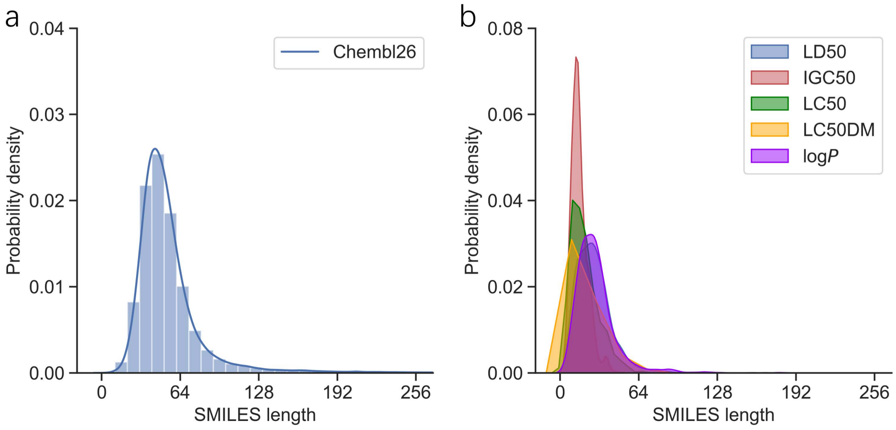

Supplementary Figure 1: The distributions of SMILES string lengths. **a**. The ChEMBL database. **b** Four toxicity datasets.

2 Supplementary Note 2

In this work, three different evaluation metrics, including the squared Pearson correlation coefficient (R^2), root mean squared error (RMSE), and mean absolute error (MAE), were used to evaluate the performances of different regression model.

The Pearson correlation coefficient is are defined as below:

$$R = \frac{\sum (x_i - \bar{x})(y_i - \bar{y})}{\sqrt{\sum (x_i - \bar{x})^2 \sum (y_i - \bar{y})^2}} \tag{1}$$

where x_i is the value of the x variable in ith sample, $\bar{x}$ is the mean of the values of the x variable, y_i is the value of the y variable in the ith sample, $\bar{y}$ is mean of the values of the y variable. The squared Pearson correlation coefficient (R^2) explains the relationship between the x variable and y variable.

The root mean squared error (RMSE) is defined as below:

$$\text{RMSE} = \sqrt{\frac{1}{n}\sum_{i=1}^{n}(y_i - \hat{y_i})^2} \tag{2}$$

where y_i and $\hat{y_i}$ are predicted value and true value of ith sample respectively.

The mean absolute error (MAE) measures the mean difference between the prediction and the true value,

$$\text{MAE} = \frac{1}{n}\sum_{i=1}^{n}|y_i - \hat{y_i}| \tag{3}$$

where y_i and $\hat{y_i}$ are predicted value and true value of ith sample respectively.

For classification task, the accuracy is simply the rate of correct classification. The receiver operating characteristic (ROC) curve is a graphical plot that illustrates the diagnostic ability of a binary classifier system as its discrimination threshold is varied. The false positive rate (FPR) and true positive rate (TPR) are used as the axis. FPR and TPR are defined as follows:

$$\text{FPR} = \frac{\text{false positive}}{\text{false positive} + \text{true negative}} \tag{4}$$

$$\text{TPR} = \frac{\text{true negative}}{\text{true negative} + \text{false negative}} \tag{5}$$

The area under the receiver operating characteristic convex hull (AUC-ROC) is used in this work to evaluate the performance of the classification model.

3 Supplementary Note 3

3.1 Input processing

In this work, all input SMILES strings for bidirectional transformer need to be processing. A total of 51 symbols, as listed in Supplementary Table 2, are used to split these SMILES strings. We add a ‘$< s >$’ symbol and a ‘$< \backslash s >$’ at the beginning and end of each input SMILES, which represent the beginning and the end of each input, respectively. Besides, the ‘$< unk >$’ is used to represent some undefined symbols. Since the length of SMILES varies from molecule to molecule, the ‘$< pad >$’ is used as a padding symbol to fill in short inputs to reach the preset length. For the self-supervised learning (SSL) -based pre-training, the 15% symbol of the input SMILES needs to be operated. Among these 15% symbols, 80% of symbols were masked, 10% of the symbols were unchanged, and the remaining 10% were randomly replaced.

3.2 Bidirectional transformer model parametrization

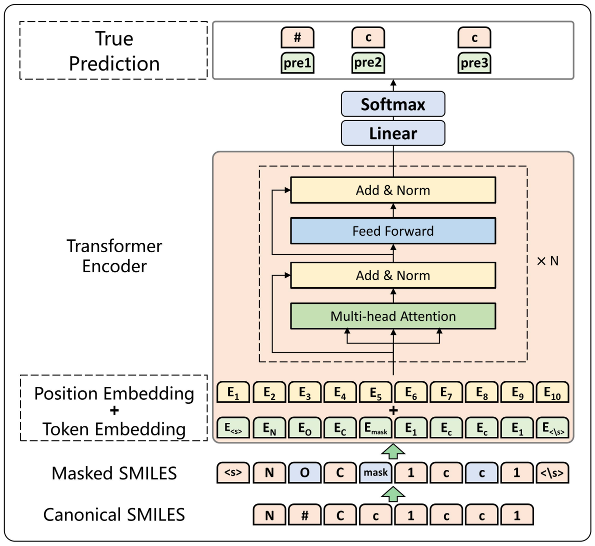

Supplementary Figure 2: The whole structure of the bidirectional encoder from transformers used in pre-training.

SSL-based pre-training Similar with the architecture of bidirectional encoder representations from transformers (BERT)[12], our pre-training model is a multi-layer bidirectional transformer encoder, as shown in Supplementary Figure 2. Each transformer layer contains two sub-layers. The first is a multi-head self-attention layer, and the second is a fully connected feed-forward neural network. The residual connection is applied to each of the two sub-layers, followed by layer normalization.[13] Each transformer layer maps the output features from the former transformer layer or the embedded features from the input into different nonlinear space. The attention mechanism used in the transformer encoder is scaled dot-product attention and it is formulated as follow,

$$\text{Attention}(Q, K, V) = \text{Softmax}(\frac{QK^T}{\sqrt{d_k}})V. \tag{6}$$

The Q, K, and V, named query matrix, key matrix, and value matrix, are mapping from input data. The dot products of the query matrix and key matrix are divides by the scaling factor $\sqrt{d_k}$, where the d_k is the embedding dimension. In practice, a multi-head self-attention mechanism is applied in the transformer encoder, where different heads could pay attention to various aspects and improve performance. On the top of the N transformer encoder layers, there is a linear layer transforming the embedding dimension into

the vocabulary size. Finally, the softmax function is used to select the maximum probability value of each masked location and report the corresponding prediction result.

The proposed model is based on Fairseq [14], which is a Sequence-to-Sequence Toolkit written in Python and PyTorch [15], and slightly modified so that it can be used for molecular analysis. In this work, the pre-training bidirectional transformer model contains 8 Transformer encoder layers, the embedding dimension is set to 512, the number of self-attention heads is 8, and the embedding size of fully connected feed-forward layers is 1024. The maximum sequence length is set to 256, including the start and terminate symbols. For better convergence, the Adam optimizer [16] is used in the pre-training and fine-tune, the Adam betas are (0.9, 0.999), and the weight decay is 0.1. Besides, a warming-up strategy is applied for the first 4000 updates and the total update steps are one million, the maximum learning rate is set to 0.0001 in this strategy. The cross-entropy was applied to measure the difference between the predicted symbols and the real symbols at the masked position. The model is trained on six Tesla V100-SXM2 GPUs and the maximum sequence number in each GPU is set to 64.

SSL-based and SL-based fine-tuning There are two strategies to be used in the fine-tuning stage: self-supervised learning (SSL) -based fine-tuning of task-specific data without using their labels and sequential supervised learning (SL) -based fine-tuning of task-specific data with their labels. For SSL-based fine-tune, the pre-trained model is fed with the input data of the downstream task-specific datasets, including both training sets and test sets. For each SMILES string, we randomly select 15% symbols to be a training-validation set in our loss function. Only 50% symbols of the set were masked and the remaining 50% symbols of the set were unchanged. A warming-up strategy is also applied for the first 500 updates. The total update steps are 2000. The maximum learning rate is set to 0.00001 in this stage. In the last hidden layer, the embedded vector of length 512 correspondings to the first special symbol $< s >$ is used for molecular property prediction. Supplementary Figure 3 shows the workflow of generating molecular fingerprints from the SSL-based fine-tuned model.

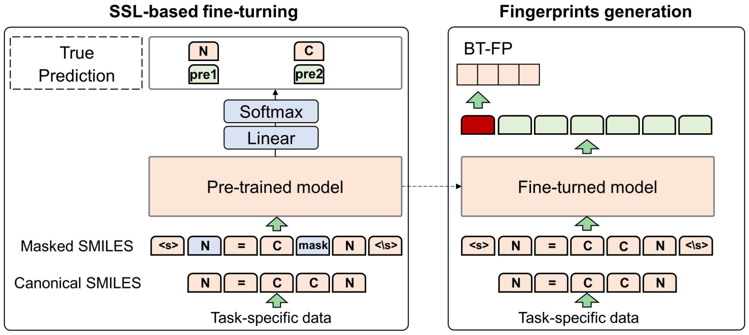

Supplementary Figure 3: Workflow for generating molecular fingerprints from the pre-trained and SSL-based fine-tuned model. Three two mask operations, 'mask' and 'no changing', are retained in the self-supervised fine-tuning stage. The labels of task-specific data are disregarded in the SSL-based fine-tuning stage. Here, $< s >$ is a special leading symbol added in front of every input SMILES, and $< \backslash s >$ is a terminating symbol. At the stage of fingerprints generation, $< s >$'s embedding vector from the bidirectional encoder is utilized to represent the molecular fingerprint (BT-FP).

For sequential SL-based fine-tuning, the labels of task-specific data are utilized. The pre-trained model will be fed with data from the training set of the task-specific dataset, and no additional 'mask' operations are required for the input SMILES. The Adam optimizer is set as the same as that of pre-training. The maximum learning rate is set to 10^{-5}. The warming-up strategy is used for the first 500 updates and the total update steps are 5000 for each dataset. The mean square error is used in this fine-tuning stage, as shown in Supplementary Figure 4. All models were trained on six Tesla V100-SXM2 GPUs and the maximum sequence number in each GPU is set to 64.

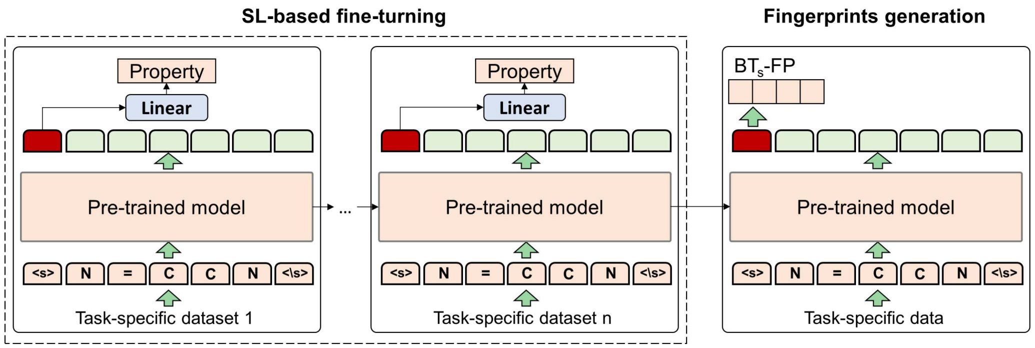

Supplementary Figure 4: Workflow for generating molecular fingerprints from the pre-trained and sequential supervised learning (SL) fine-tuned model. Labeled task-specific data are employed in the sequential SL-based fine-tuning stage. At the stage of fingerprints generation, $< s >$'s embedding vector before last linear layer is utilized to represent the molecular fingerprint (BT_s-FP).

3.3 Algebraic graph model parametrization

In order to select a general AG-FP for all four toxicity data sets, we need to combine the kernel function and graph matrix type properly. For the sake of convenience, we use the notation $\text{AG}^{\mathcal{M}}_{\Omega,\beta,\tau}$, to indicate the AG-FPs generated by using interactive matrix type $\mathcal{M}$ with kernel function ω and corresponding kernel parameters β and τ. Here, $\mathcal{M} = \{Adj, Lap\}$ represents a set of adjacency matrix and Laplacian matrix. $\omega = \{E, L\}$ refers to a set of generalized exponential and generalized Lorentz kernels. In addition, the kernel parameter $\beta = \kappa$ if $\omega = E$, and $\beta = \upsilon$ if $\omega = L$. And τ is used such that $\eta_{k_1k_2} = \tau(\bar{r}_{k_1} + \bar{r}_{k_2})$, where $\bar{k}_{k_1}$ and $\bar{r}_{k_2}$ are the van der Waals radii of element type k_1 and k_2, respectively. Kernel parameters β and τ as selected based on the cross validation with a random split of the training data. It has been shown that multiscale information can boost the performance of predictor. [17, 18] In this work, we consider at most two kernels. As a straightforward notation extension, two kernels can be parametrized by $\text{AG}^{\mathcal{M}_1,\mathcal{M}_2}_{\omega_1,\beta_1,\tau_1;\Omega_2,\beta_2,\tau_2}$. To attain the best performance using AG-FP, the kernel parameters need to be optimized. We vary β, both τ and κ, from 0.5 to 6 with an increment of 0.5, while τ values are chosen from 0.5 to 6 with an increment of 0.5. The high values of the power order such as $\beta \in \{10, 15, 20\}$ are also considered to approximate the idea low-pass filter.[19] We use the method of 5-fold cross-validation (CV) to select the kernel hyperparameters $\mathcal{M}$, Ω, β and τ. Supplementary Figure 5**a** shows the CV results of the single-kernel model ($\text{AG}^{\mathcal{M}_1}_{\omega_1,\beta_1,\tau_1}$), and R^2 is used as the evaluation metrics. Then based on the optimal kernel parameters in the single-kernel model, the two-kernel model, $\text{AG}^{\mathcal{M}_1,\mathcal{M}_2}_{\omega_1,\beta_1,\tau_1;\Omega_2,\beta_2,\tau_2}$, can be optimized by using 5-fold CV on training sets. Supplementary Figure 5**b** in the supplement material reports the best models with associated R^2 in this experiment. All cross-validations were performed for toxicity training sets, and the scores were based on the mean value of R^2 in the four training sets.

For the toxicity and log$P$ datasets, there are 10 commonly occurring element types, i.e., {H, C, N, O, F, P, S, Cl, Br, I}, which means 100 element interactive pairs will form based on the combinations of these 10 element types in molecules. For adjacency matrices, only positive eigenvalues are considered. Note that Laplacian matrices are positive semidefinite. As discussed in the Methods section, we can compute nine descriptive statistical values, namely the maximum, minimum, average, summation, median, standard deviation, and variance of all eigenvalues. Another two values are the number of considered eigenvalues and the sum of the second power of eigenvalues. This gives rise to a total of 900 features for one kernel, which means that we can get an 1800 dimension AG-FP for each molecule if we use two-kernel information. The optimal two-kernel algebraic graph models are $\text{AG}^{\text{Lap,Lap}}_{L,10,0.5;L,20,0.5}$, and the average R^2 of all four toxicity datasets is 0.631.

For the partition coefficient dataset, we also use two-kernel information as the final AG-FPs. There are

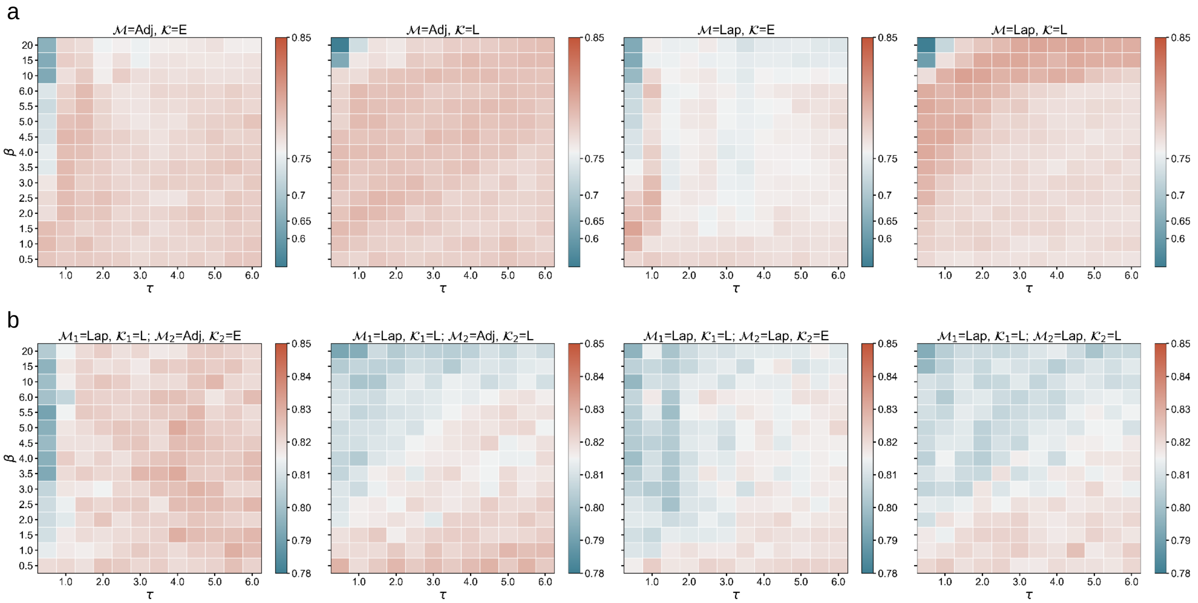

Supplementary Figure 5: Squared Pearson correlation coefficients (R^2) from 5-fold cross-validation of $\text{AG}^{\mathcal{M}}_{\Omega,\beta,\tau}$, and $\text{AG}^{\mathcal{M}_1,\mathcal{M}_2}_{\omega_1,\beta_1,\tau_1;\Omega_2,\beta_2,\tau_2}$ on the training data of four toxicity datasets are plotted against different values of τ and β. **a**. The best hyperparameters and R^2 for these one-scale models are found to be ($\text{AG}^{\text{Adj}}_{E,1.5,0.5}$, average $R^2 = 0.616$), ($\text{AG}^{\text{Adj}}_{L,4.5,0.5}$, average $R^2 = 0.616$), ($\text{AG}^{\text{Lap}}_{E,5.5,0.5}$, average $R^2 = 0.610$) and ($\text{AG}^{\text{Lap}}_{L,10,0.5}$, average $R^2 = 0.620$) from left to right separately. **b**. Based on the best one-scale model, the best hyperparameters and R^2 for these multiscale models are found to be ($\text{AG}^{\text{Lap,Adj}}_{L,10,0.5;E,6,0.5}$, average $R^2 = 0.628$), ($\text{AG}^{\text{Lap,Adj}}_{L,10,0.5;L,20,0.5}$, average $R^2 = 0.629$), ($\text{AG}^{\text{Lap,Lap}}_{L,10,0.5;E,6,0.5}$, average $R^2 = 0.627$) and ($\text{AG}^{\text{Lap,Lap}}_{L,10,0.5;E,20,0.5}$, average $R^2 = 0.631$) from left to right separately.

the same 10 element types as for toxicity datasets and there are also 100 element interactive pairs that will form in each molecule. As shown in Supplementary Figure 6a, we firstly selected the best hyperparameters for these one-scale models, which are $\text{AG}^{\text{Adj}}_{E,4.5,1.0}$ ($R^2 = 0.798$), $\text{AG}^{\text{Adj}}_{L,2.0,1.0}$ ($R^2 = 0.799$), $\text{AG}^{\text{Lap}}_{E,1.5,1.0}$ ($R^2 = 0.81$), and $\text{AG}^{\text{Lap}}_{L,10,1.5}$ ($R^2 = 0.811$). Based on the optimal one-scale model ($\text{AG}^{\text{Lap}}_{L,10,1.5}$), the best multiscale model is found to be $\text{AG}^{\text{Lap,Adj}}_{L,10,1.5;E,5,4}$, as shown in Supplementary Figure 6b, the value of R^2 is 0.831. The gradient boosting decision tree (GBDT) is used to select optimal algebraic graph model hyperparameters. The parameters of GBDT vary with the size of the training set, which are listed in Supplementary Table 3.

For the FreeSolv dataset, we use two-kernel information as the final AG-FPs. There are the same 10 element types as for toxicity datasets and there are also 100 element interactive pairs that will be constructed for each molecule. As shown in Supplementary Figure 7a, we firstly select the best hyperparameters for these one-scale models. Based on the optimal one-scale model ($\text{AG}^{\text{Adj}}_{L,10.0,0.5}$, $R^2 = 0.92$), the best multiscale model is found to be $\text{AG}^{\text{Adj,Adj}}_{E,6.0,0.5;E,6,0.5}$, as shown in Supplementary Figure 7b, the value of R^2 is 0.935. The gradient boosting decision tree (GBDT) is used to select optimal algebraic graph model hyperparameters. The parameters of GBDT vary with the size of the training set, which are listed in Supplementary Table 3.

For the lipophilicity dataset, we use two-kernel information as the final AG-FPs. There are the same 10 element types as for toxicity datasets and there are also 100 element interactive pairs that will form in each molecule. As shown in Supplementary Figure 8a, we firstly selected the best hyperparameters for these one-scale models. Based on the optimal one-scale model ($\text{AG}^{\text{Lap}}_{E,3.5,0.5}$, $R^2 = 0.672$), the best multiscale model is found to be $\text{AG}^{\text{Lap,Lap}}_{E,3.5,0.5;E,2.5,0.5}$, as shown in Supplementary Figure 8**b**, the value of R^2 is 0.688. The gradient boosting decision tree (GBDT) is used to select optimal algebraic graph model hyperparameters. The parameters of GBDT vary with the size of the training set, which are listed in Supplementary Table 3.

For the BBBP dataset, we still use two-kernel information as the final AG-FPs. The deviation (DEV)

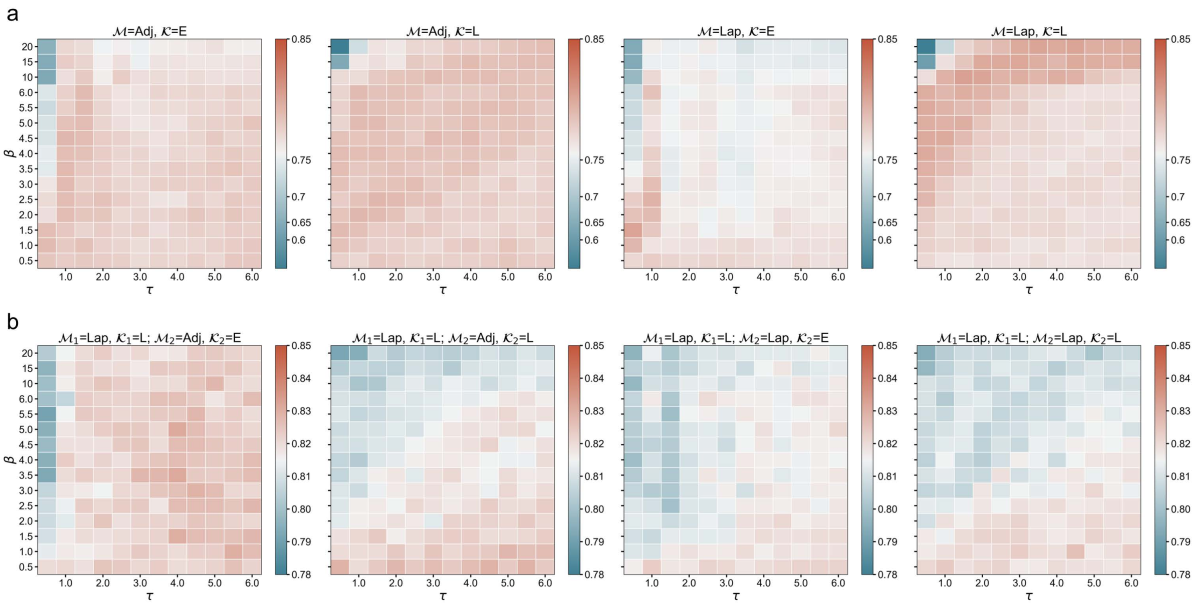

Supplementary Figure 6: Squared Pearson correlation coefficients (R^2) from 5-fold cross-validation of $\mathrm{AG}^{\mathcal{M}}_{\Omega,\beta,\tau}$, and $\mathrm{AG}^{\mathcal{M}_1,\mathcal{M}_2}_{\omega_1,\beta_1,\tau_1;\Omega_2,\beta_2,\tau_2}$ on the training data of partition coefficient data sets are plotted against different values of τ and β. **a**. The best hyperparameters and R^2 for these one-scale models are found to be ($\mathrm{AG}^{\mathrm{Adj}}_{E,4.5,1.0}$, $R^2 = 0.798$), ($\mathrm{AG}^{\mathrm{Adj}}_{L,2.0,1.0}$, $R^2 = 0.799$), ($\mathrm{AG}^{\mathrm{Lap}}_{E,1.5,1.0}$, $R^2 = 0.81$) and ($\mathrm{AG}^{\mathrm{Lap}}_{L,10,1.5}$, $R^2 = 0.811$) from left to right separately. **b**. Based on the best one-scale model, the best hyperparameters and R^2 for these multiscale models are found to be ($\mathrm{AG}^{\mathrm{Lap,Adj}}_{L,10,1.5;E,5,4}$, $R^2 = 0.831$), ($\mathrm{AG}^{\mathrm{Lap,Adj}}_{L,10,1.5;L,0.5,5.5}$, $R^2 = 0.829$), ($\mathrm{AG}^{\mathrm{Lap,Lap}}_{L,10,1.5;E,0.5,1}$, $R^2 = 0.823$) and ($\mathrm{AG}^{\mathrm{Lap,Lap}}_{L,10,1.5;E,1,4.5}$, $R^2 = 0.826$) from left to right separately.

is used in parameter selection. There are the same 10 element types as for toxicity datasets and there are also 100 element interactive pairs will form in each molecule. As shown in Supplementary Figure 9a, we firstly selected the best hyperparameters for these one-scale models. Based on the optimal one-scale model ($\mathrm{AG}^{\mathrm{Lap}}_{L,20,0.5}$, $DEV = 0.797$), the best multiscale model is found to be $\mathrm{AG}^{\mathrm{Lap,Adj}}_{L,20,0.5;L,20,0.5}$, as shown in Supplementary Figure 9b, the value of DEV is 0.795. The gradient boosting decision tree (GBDT) is used to select optimal algebraic graph model hyperparameters. The parameters of GBDT vary with the size of the training set, which are listed in Supplementary Table 3.

3.4 Feature fusion

Based on a large amount of unlabeled data, BT-FP can capture the overall information of molecules after pre-training and fine-tuning. AG-FP, on the other hand, as insight based on physical and chemical knowledge, can obtain more detailed information of molecular structure, including dihedral angle and relative distance of atoms, with the help of algebraic graph theory. The proposed AGBT-FP in this work is a fusion of BT-FP and AG-FP. The random forest (RF) is used to fuse BT-FP and AG-FP. First, we combine BT-FP and AG-FP. Then the RF algorithm was used to select top 512 features. The parameters of RF vary with the size of the training set. All parameters are listed in Supplementary Table 3. The final AGBT-FP's dimension is set to 512, which is the same as that for BT-FP's.

3.5 Downstream machine learning algorithms

To compare the AGBT and other fingerprints' performance on specific tasks, three machine learning algorithms are used: gradient boosting decision tree (GBDT), single-task deep neural network (ST-DNN),

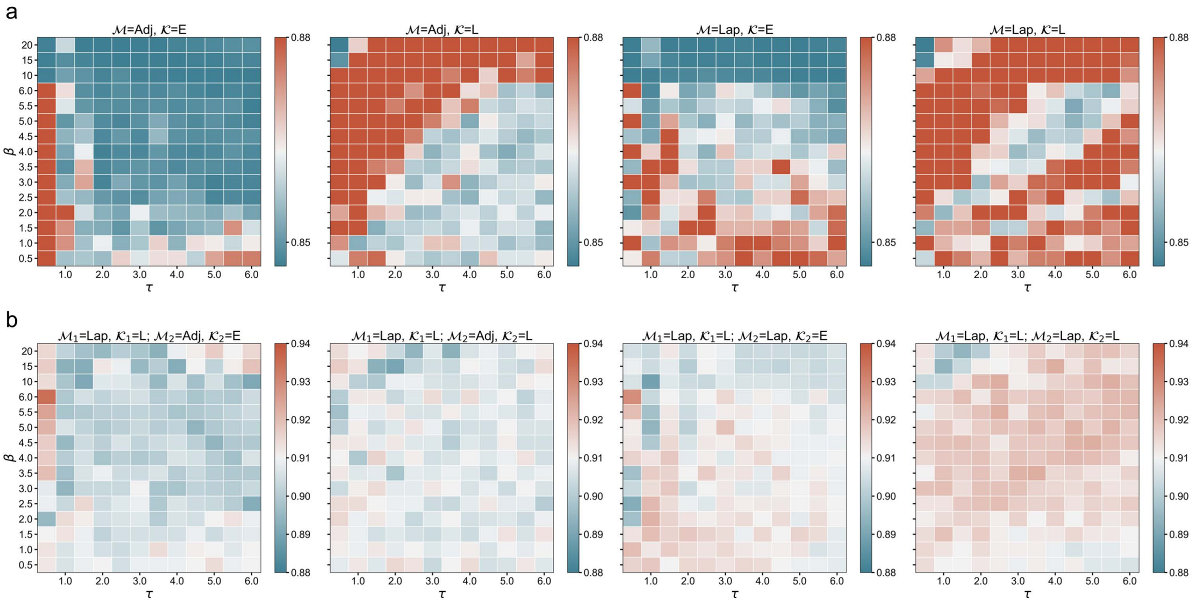

Supplementary Figure 7: Squared Pearson correlation coefficients (R^2) from 5-fold cross-validation of $\mathrm{AG}^{\mathcal{M}}_{\Omega,\beta,\tau}$, and $\mathrm{AG}^{\mathcal{M}_1,\mathcal{M}_2}_{\omega_1,\beta_1,\tau_1;\Omega_2,\beta_2,\tau_2}$ on the FreeSolv data sets are plotted against different values of τ and β. **a**. The best hyperparameters and R^2 for these one-scale models are found to be ($\mathrm{AG}^{\mathrm{Adj}}_{E,6.0,0.5}$, $R^2 = 0.907$), ($\mathrm{AG}^{\mathrm{Adj}}_{L,10.0,0.5}$, $R^2 = 0.92$), ($\mathrm{AG}^{\mathrm{Lap}}_{E,2.5,1.0}$, $R^2 = 0.899$) and ($\mathrm{AG}^{\mathrm{Lap}}_{L,4.5,0.5}$, $R^2 = 0.899$) from left to right separately. **b**. Based on the best one-scale model, the best hyperparameters and R^2 for these multiscale models are found to be ($\mathrm{AG}^{\mathrm{Adj,Adj}}_{E,6.0,0.5;E,6,0.5}$, $R^2 = 0.935$), ($\mathrm{AG}^{\mathrm{Adj,Adj}}_{E,6.0,0.5;L,15,0.5}$, $R^2 = 0.917$), ($\mathrm{AG}^{\mathrm{Adj,Lap}}_{E,6.0,0.5;E,6,0.5}$, $R^2 = 0.929$) and ($\mathrm{AG}^{\mathrm{Adj,Lap}}_{E,6.0,0.5;E,3.5,10}$, $R^2 = 0.923$) from left to right separately.

and multitask deep neural network (MT-DNN).

Gradient boosting decision tree (GBDT). GBDT is a robust machine learning regressor. In this approach, individual decision trees are successively combined in a stage-wise fashion to achieve the capability of learning complex features. It uses both gradient and boosting strategies to reduce model errors. Compared to the deep neural network (DNN) approaches, this ensemble method is robust, relatively insensitive to hyperparameters, and easy to implement. Moreover, they are much faster to train than DNN. In fact, for small data sets, GBDT can perform even better than DNN or other deep learning algorithms.[20, 21] Therefore, GBDT has been applied to a variety of QSAR prediction problems, such as toxicity, solvation, and binding affinity predictions.[22, 23]

The GBDT is used to predict the toxicity and $\log P$ in this work and implemented by the scikit-learn package.[24] In this work, there are five data sets with their training data size varying from 283 to 8199. To better compare feature performance, we set only two sets of parameters according to the size of the training set for GBDT. The detailed values of these hyperparameters are given in Supplementary Table 3.

Single-task deep neural network (ST-DNN). A DNN mimics the learning process of a biological brain by constructing a wide and deep architecture of numerous connected neuron units. A typical deep neural network often includes multiple hidden layers. In each layer, there are hundreds or even thousands of neurons. During learning stages, weights on each layer are updated by backpropagation. With a complex and deep network, DNN is capable of constructing hierarchical features and model complex nonlinear relationships. ST-DNN is a regular deep learning algorithm. It only takes care of one single prediction task. Therefore, it only learns from one specific training dataset. A typical four-layer ST-DNN is showed in Supplementary Figure 10a, where N_i ($i = 1, ..., 4$), represents the number of neurons in the ith hidden layer.

Multitask deep neural network (MT-DNN). The multitask (MT) learning technique has achieved

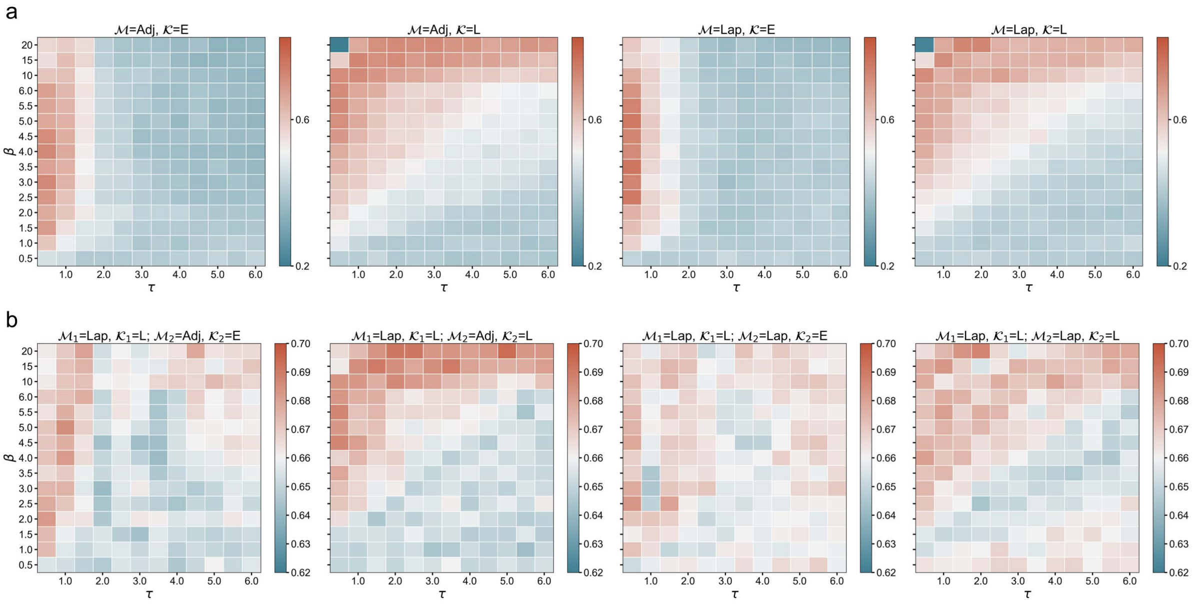

Supplementary Figure 8: Squared Pearson correlation coefficients (R^2) from 5-fold cross-validation of $\mathrm{AG}^{\mathcal{M}}_{\Omega,\beta,\tau}$, and $\mathrm{AG}^{\mathcal{M}_1,\mathcal{M}_2}_{\omega_1,\beta_1,\tau_1;\Omega_2,\beta_2,\tau_2}$ on the Lipophilicity data sets are plotted against different values of τ and β. **a**. The best hyperparameters and R^2 for these one-scale models are found to be ($\mathrm{AG}^{\mathrm{Adj}}_{E,4.0,0.5}$, $R^2 = 0.657$), ($\mathrm{AG}^{\mathrm{Adj}}_{L,15.0,1}$, $R^2 = 0.659$), ($\mathrm{AG}^{\mathrm{Lap}}_{E,3.5,0.5}$, $R^2 = 0.672$) and ($\mathrm{AG}^{\mathrm{Lap}}_{L,20,2}$, $R^2 = 0.659$) from left to right separately. **b**. Based on the best one-scale model, the best hyperparameters and R^2 for these multiscale models are found to be ($\mathrm{AG}^{\mathrm{Lap,Adj}}_{E,3.5,0.5;E,5,1}$, $R^2 = 0.685$), ($\mathrm{AG}^{\mathrm{Lap,Adj}}_{E,3.5,0.5;L,20,5}$, $R^2 = 0.694$), ($\mathrm{AG}^{\mathrm{Lap,Lap}}_{E,3.5,0.5;E,2.5,0.5}$, $R^2 = 0.683$) and ($\mathrm{AG}^{\mathrm{Lap,Lap}}_{E,3.5,0.5;E,2.5,0.5}$, $R^2 = 0.688$) from left to right separately.

much success in qualitative Merck and Tox21 prediction challenges.[25, 26, 27] In the MT framework, multiple tasks share the same hidden layers. However, the output layer is attached to different tasks. This framework enables the neural network to learn all the data simultaneously for different tasks. Thus, the commonalities and differences among various data sets can be exploited. It has been shown that MT learning typically can improve the prediction accuracy of relatively small data sets if it combines with relatively larger data sets in its training. Supplementary Figure 10b is an illustration of a typical four-layer MT-DNN for training four different tasks simultaneously. Suppose there are totally T tasks and the training data for the tth task are $(X_i^t, y_i^t)_{i=1}^{N_t}$, where $t = 1, ..., T$, $i = 1, ..., N_t$, where N_t is the number of samples in the tth task, and X_i^t is the feature vector for the ith sample in the tth task, y_i^t is the label value of the ith sample in the tth task, respectively. The purpose of MT learning is to simultaneously minimize the loss function:

$$argmin \sum_{t=1}^{T} \sum_{i=1}^{N_t} L(y_i^t, f^t(X_i^t, \theta^t)), \tag{7}$$

where f^t is the prediction for the ith sample in the tth task by our MT-DNN, which is a function of the feature vector X_i^t, L is the loss function, and θ^t is the collection of machine learning hyperparameters. A popular cost function for regression is the mean squared error, which is formulated as:

$$L(y_i^t, f^t(X_i^t, \theta^t)) = \frac{1}{N_t} \sum_{i=q}^{N_t} (y_i^t - f^t(X_i^t, \theta^t))^2. \tag{8}$$

In this work, MT-DNN is only applied to predict the toxicity. The ultimate goal of MT-DNN learning is to potentially improve the overall performance of multiple toxicity prediction models, especially for the smallest dataset that performs relatively poorly in the ST-DNN. More concretely, it is reasonable to assume

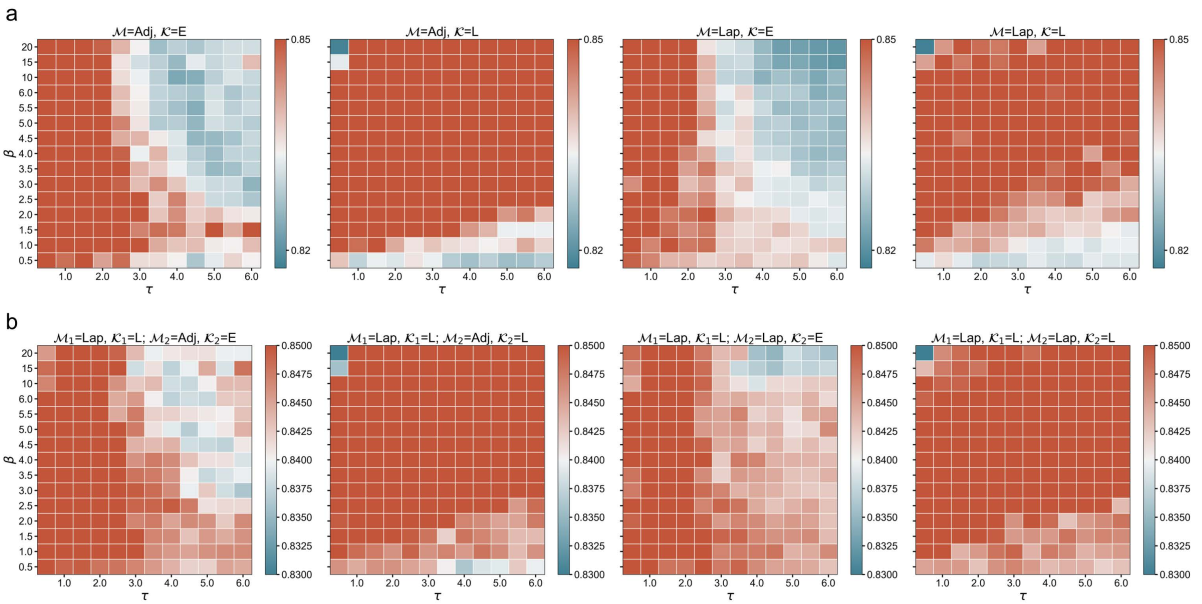

Supplementary Figure 9: The deviation (DEV) from 5-fold cross-validation of $\mathrm{AG}^{\mathcal{M}}_{\Omega,\beta,\tau}$, and $\mathrm{AG}^{\mathcal{M}_1,\mathcal{M}_2}_{\omega_1,\beta_1,\tau_1;\Omega_2,\beta_2,\tau_2}$ on the BBBP data sets are plotted against different values of τ and β. **a**. The best hyperparameters and DEV for these one-scale models are found to be ($\mathrm{AG}^{\mathrm{Adj}}_{E,10,4}$, $DEV = 0.829$), ($\mathrm{AG}^{\mathrm{Adj}}_{L,20,0.5}$, $DEV = 0.804$), ($\mathrm{AG}^{\mathrm{Lap}}_{E,15,6}$, $DEV = 0.823$) and ($\mathrm{AG}^{\mathrm{Lap}}_{L,20,0.5}$, $DEV = 0.797$) from left to right separately. **b**. Based on the best one-scale model, the best hyperparameters and DEV for these multiscale models are found to be ($\mathrm{AG}^{\mathrm{Lap,Adj}}_{L,20,0.5;E,3,6}$, $DEV = 0.836$), ($\mathrm{AG}^{\mathrm{Lap,Adj}}_{L,20,0.5;L,20,0.5}$, $DEV = 0.795$), ($\mathrm{AG}^{\mathrm{Lap,Lap}}_{L,20,0.5;E,20,4.5}$, $DEV = 0.835$) and ($\mathrm{AG}^{\mathrm{Lap,Lap}}_{L,20,0.5;E,20,0.5}$, $DEV = 0.796$) from left to right separately.

that different toxicity indices share a common statistic pattern so that these different tasks can be trained simultaneously when their feature vectors are constructed in the same manner. For our toxicity prediction, four different tasks (LD50, IGC50, LC50, LC50DM data sets) are trained together. This leads to four output neurons in the output layer, with each neuron being specific to one of four tasks.

The performance of deep neural network models depends on their architecture, input data dimension, and hyperparameters. For BT-FP and AGBT-FP, the feature sizes are both 512, which means that the network with the same architecture can be used to train these two sets of features. The input layer contains 512 neurons, followed by four hidden layers with 1024, 512, 512, and 512 neurons, respectively. For the present regression problem, only one neuron in the final output layer. For AG-FP, it contains 1800 features, and thus a more complex network structure is required. In this case, we set 1800 neurons in the input layer, followed by 5 hidden layers with 2048, 1024, 512, 512, and 512 neurons, respectively. The output layer has one neuron. Other network parameters are all the same for these three kinds of molecular features. The stochastic gradient descent (SGD) with a momentum of 0.5 is used as an optimizer. We use 2000 epochs to train all the networks. The mini-batch size is set to 8. The learning rate is set to 0.01 in the first 1000 epochs and 0.001 for the rest epochs. These hyperparameters are applied to both ST-DNN and MT-DNN. All the DNN models are built and trained in Pytorch.[15]

Supplementary Table 3: RF and GBDT parameters for different toxicity training-set sizes

Training-set Szie	RF Parameters	GBDT Parameters
> 1000	n_estimators = 10000	n_estimators = 10000
	criterion = 'mse'	max_depth = 8
	max_depth = 8	min_samples_split = 4
	min_samples_split = 4	learning_rate = 0.01
	min_samples_leaf = 1	subsample = 0.3
	min_weight_fraction_leaf = 0.0	max_features='sqrt'
< 1000	n_estimators = 10000	n_estimators = 10000
	criterion = 'mse'	max_depth = 7
	max_depth = 7	min_samples_split = 3
	min_samples_split = 3	learning_rate = 0.01
	min_samples_leaf = 1	subsample = 0.2
	min_weight_fraction_leaf = 0.0	max_features='sqrt'

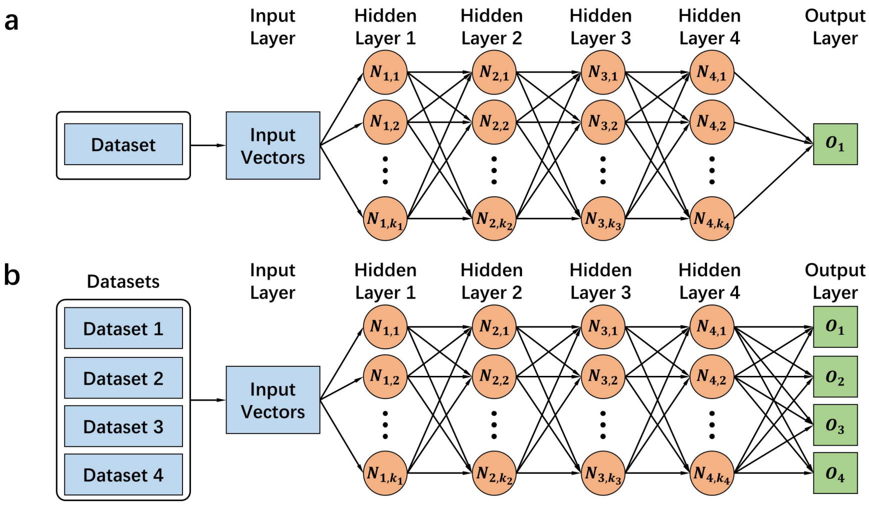

Supplementary Figure 10: ST-DNN and MT-DNN framework. a) An illustration of a typical ST-DNN. Only one dataset is trained in this network. Four hidden layers are included, k_i (i = 1, 2, 3, 4) represents the number of neurons in the ith hidden layer and $N_{i,j}$ is the jth neuron in the ith hidden layer. Here, O_1 is the single output for the model. b) An illustration of a typical MT-DNN training four tasks (datasets) simultaneously. Four hidden layers are included in this network, k_i (i = 1, 2, 3, 4) represents the number of neurons in the ith hidden layer and $N_{i,j}$ is the jth neuron in the ith hidden layer. Here O_1 to O_4 represent four predictor outputs for four tasks.

4 Supplementary Note 4

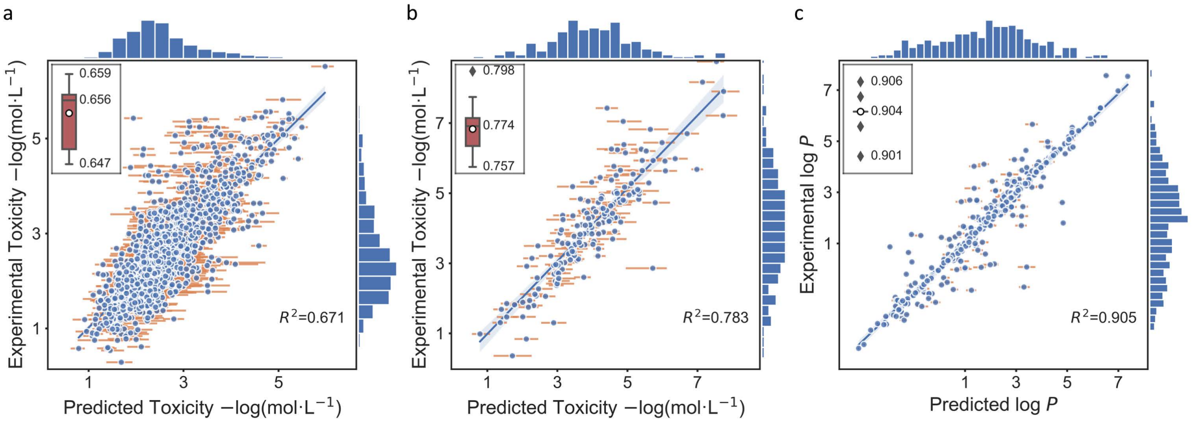

Supplementary Figure 11: Data and results of AGBT. **a**, Predicted results of AGBT-FPs with MT-DNN model for LD50 dataset (R^2=0.671, RMSE=0.554 log(mol/L)). The box plots statistic R^2 values for n=1482 independent samples examined over 20 independent machine learning experiments. **b**, Predicted results of BT-FPs with MT-DNN model for LC50 dataset (R^2=0.783, RMSE=0.692 log(mol/L)). The box plots statistic R^2 values for n=164 independent samples examined over 20 independent machine learning experiments. **c**, Predicted results of AGBT$_s$-FPs with MT-DNN model for LC50 dataset (R^2=0.905, RMSE=0.615 log(mol/L)). The box plots statistic R^2 values for n=406 independent samples examined over 20 independent machine learning experiments. The detail statistic values of box plots are listed in Supplementary Table 5

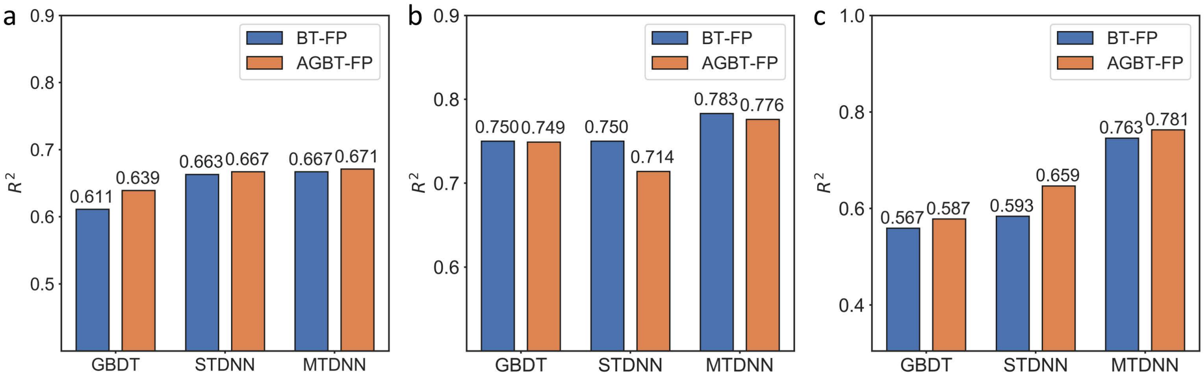

Supplementary Figure 12: Consensus R^2 values of BT-FP and AGBT-FP predictions on three machine learning algorithms, GBDT, STDNN, and MTDNN. **a** LD50 dataset, **b** LC50 dataset, **c** LC50DM dataset.

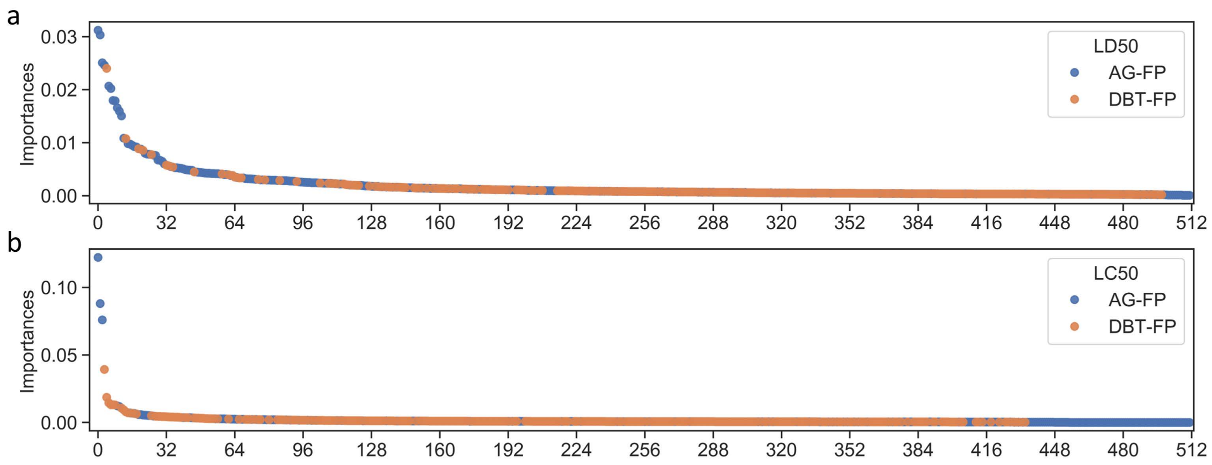

Supplementary Figure 13: The AGBT-FPs of the LD50 and LC50 datasets were ranked by their feature importance.**a** Sorted feature importance for the LD50 dataset. The top three features are from AG-FP. **b** Sorted feature importance for the LC50 dataset. The top three features are from AG-FP. For both datasets, 188/512 of the AGBT features are from AG-FPs and the remaining 348/512 are from BT-FPs.

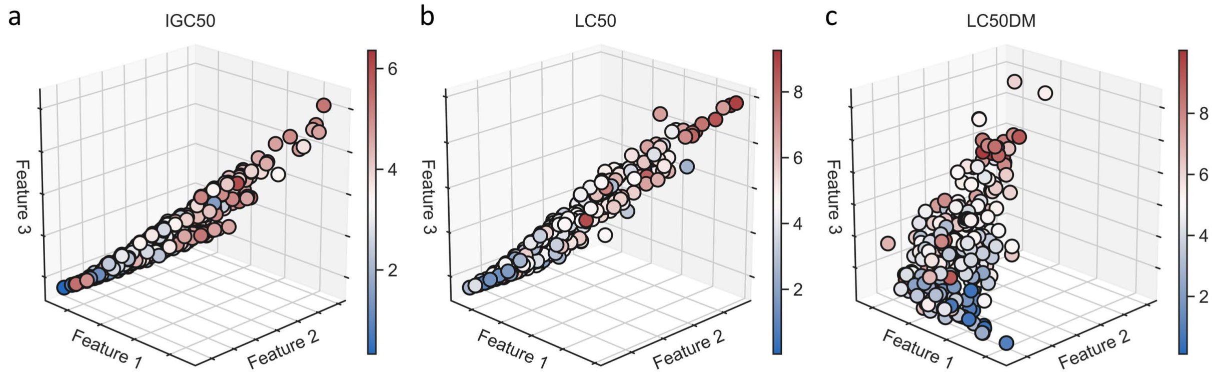

Supplementary Figure 14: Distribution of molecules in the three most important features of AGBT-FP. **a** The distribution of the IGC50 dataset. **b** The distribution of the LC50 dataset. **c** The distribution of the LC50DM dataset.

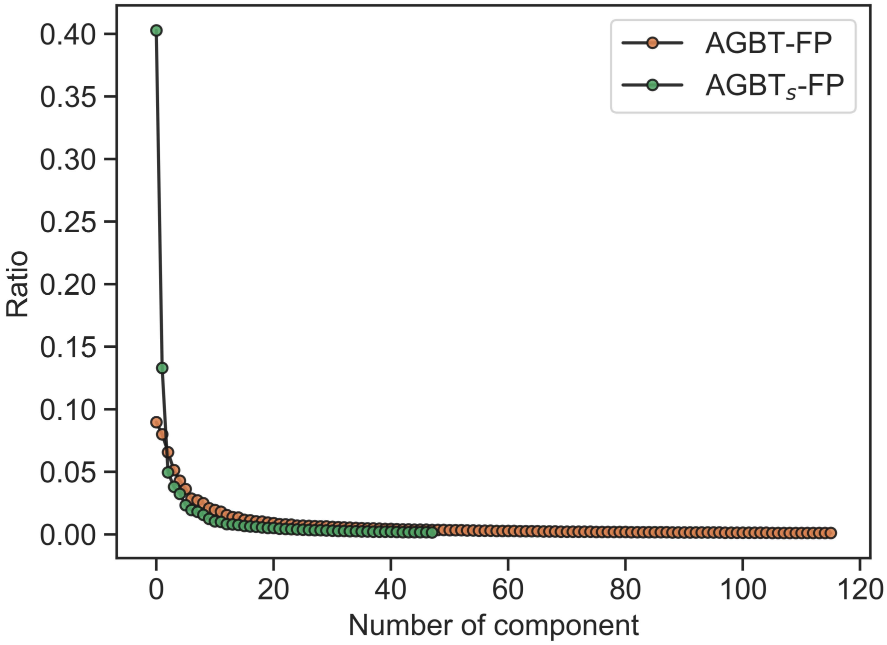

Supplementary Figure 15: The ratio refers to the rate of variability (variance) of the data explained by each principal component through principal component analysis (PCA). For AGBT-FP (orange), the first 112 components are needed to represent 90% variance, whereas for AGBT$_s$-FP (green), only the first 48 components are needed to represent 90% of the variance.

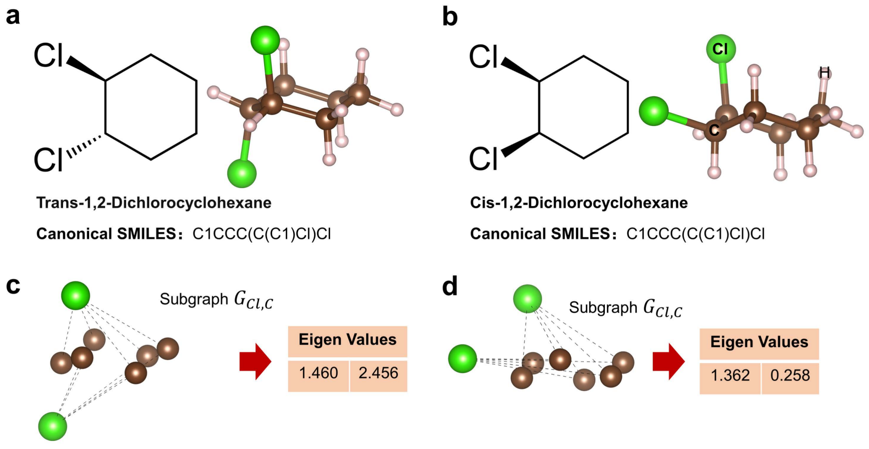

Supplementary Figure 16: Application of algebraic graph theory methods to the analysis of cis-trans structures. **a** and **b** Illustration of Trans-1,2-Dichlorocyclohexane and Cis-1,2-Dichlorocyclohexane, these two molecules share the same canonical SMILES. **c** and **d** The trans- and cis- molecular subgraph $G_{\mathrm{Cl,C}}$ for the conditions $\mathrm{AG}_{E,1.0,1.0}^{\mathrm{Adj}}$.

5 Supplementary Note 5

Tanimoto coefficient, $S_{A,B}$, is used in this work to calculate the degree of similarity between two molecules. A higher average $S_{A,B}$ of the two datasets implies a higher similarity. Tanimoto coefficient, $S_{A,B}$, is defined as follow:

$$S_{A,B} = \frac{\sum_{i=1}^{N} x_{iA} x_{iB}}{\sum_{i=1}^{N} x_{iA}^2 + \sum_{i=1}^{N} x_{iB}^2 - \sum_{i=1}^{N} x_{iA} x_{iB}}. \tag{9}$$

In this study, the similarity between the largest dataset LD50, which contains 7413 molecules, with other three datasets are list in Supplementary Table 4

Supplementary Table 4: Similarity between the Largest Dataset LD50 with the other three datasets[a]

Fingerprints	IGC50(1792)	LC50(823)	LC50DM(353)
Estate2	0.964	0.973	0.982
FP2	0.886	0.928	0.941

[a] The number in the bracket is the total size of the dataset.

Supplementary Table 5: The detail statistical values for box plots in Figure 2f, Supplementary Figure 11a, b. The box plots statistic R^2 values for n=1482 (LD50), 358 (IGC50), n=164 (LC50), n=70 (LC50DM), and n=406 (LogP) independent samples examined over 20 independent machine learning experiments.

Datasets	LD50	IGC50	LC50	LC50DM	LC50DM
Minima	0.647	0.818	0.757	0.817	0.901
Maxima	0.659	0.839	0.798	0.84	0.906
Median	0.656	0.829	0.774	0.83	0.904
1st quartile	0.649	0.827	0.766	0.824	0.904
3rd quartile	0.656	0.836	0.778	0.832	0.904
Average	0.654	0.831	0.773	0.829	0.904

Supplementary Table 6: Comparison of the reported R^2 of various predicting methods on the LD50, LC50, IGC50, LC50DM, and FDA Approved Small-Molecule, data sets.

LD50		LC50		FDA	
Method	R^2	Method	R^2	Method	R^2
AGBT-FP	0.671	AGBT-FP	0.776/0.783[a]	$AGBT_s$-FP	0.905
MACCS[20]	0.643	BTAMDL2[21]	0.750	ESTD-1[28]	0.893
FP2[20]	0.631	ESTDS[3]	0.745	Estate2[20]	0.893
HybridModel[29]	0.629	Daylight-MTDNN[20]	0.724	XLOGP3[6]	0.872
Daylight[20]	0.624	Hierarchical[29]	0.710	Estate1[20]	0.870
BESTox[30]	0.619	Single Model[22]	0.704	MACCS[20]	0.867
BTAMDL1[21]	0.605	Estate1 MTDNN[20]	0.694	ECFP[20]	0.857
Estate1[20]	0.605	Group contribution[29]	0. 686	ESTD-2[28]	0.848
Estate2[20]	0.589	HybridModel[29]	0.678	XLOGP3-AA[6]	0.847
ECFP[20]	0.586	Estate2[20]	0.662	CLOGP[6]	0.838
Hierarchical[22]	0.578	FDA[20]	0.626	Daylight[20]	0.819
Nearest neighbor[22]	0.557	FP2[20]	0.609	TOPKAT[6]	0.815
FDA[22]	0.557	MACCS[20]	0.608	xlogp2[6]	0.800
Pharm2D[20]	0.443	ECFP[20]	0.573	alogp98[6]	0.777
ERG[20]	0.392	Pharm2D[20]	0.528	KOWWIN[6]	0.771
		ERG[20]	0.348	HINT[20]	0.491
IGC50		LC50DM			
Method	R^2	Method	R^2		
AGBT-FP	0.842	$AGBT_s$-FP	0.830		
HybridModel[29]	0.81	HybridModel[29]	0.616		
Hierarchical[22]	0.719	Hierarchical[22]	0.695		
FDA[29]	0.747	Single model[29]	0.697		
GroupContr.[29]	0.682	FDA[29]	0.565		
NearestNei.[29]	0.6	GroupContr.[29]	0.671		
Daylight-BTAMDL1[21]	0.724	NearestNei.[29]	0.733		
Estate2[20]	0.742	Daylight-BTAMDL2[21]	0.700		
Estate1[20]	0.735	Estate2[20]	0.623		
Daylight[20]	0.717	Estate1[20]	0.684		
FP2[20]	0.681	Daylight[20]	0.694		
ECFP[20]	0.647	FP2[20]	0.357		
MACCS[20]	0.643	ECFP[20]	0.452		
Pharm2D[20]	0.384	MACCS[20]	0.434		
ERG[20]	0.274	Pharm2D[20]	0.275		
DG-GL[31]	0.781	ERG[20]	0.336		

[a] only BT-FP is used as input;

Supplementary Table 7: Performance of descriptors generated with the AGBT framework on 8 datasets. The 5 descriptors generated by our method, AG-FP, BT-FP, BT_s-FP, ABGT-FP, $AGBT_s$-FP obtained, 1, 3, 4, 8, 7 best scores on 8 datasets for 23 evaluation metrics, respectively.*

Datasets	LD50			IGC50			LC50		
Metric	R^2	RMSE	MAE	R^2	RMSE	MAE	R^2	RMSE	MAE
AG-FP	0.647[a]	0.573[a]	0.423[a]	0.788[d]	0.454[d]	0.315[d]	0.713[d]	0.786[d]	0.535[d]
BT-FP	0.667[d]	0.557[d]	0.406[d]	0.839[d]	0.395[d]	0.284[d]	**0.783**[d]	**0.692**[d]	0.492[d]
BT_s-FP	0.617[d]	0.602[d]	0.434[d]	0.798[d]	0.445[d]	0.313[d]	0.75[d]	0.734[d]	0.53[d]
AGBT-FP	**0.671**[d]	**0.554**[d]	**0.401**[d]	**0.842**[d]	**0.391**[d]	**0.273**[d]	0.776[d]	0.703[d]	**0.491**[d]
$AGBT_s$-FP	0.612[d]	0.606[d]	0.435[d]	0.805[d]	0.437[d]	0.304[d]	0.75[d]	0.734[d]	0.525[d]
Datasets	LC50DM			LogP			FreeSolv		
Metric	R^2	RMSE	MAE	R^2	RMSE	MAE	R^2	RMSE	MAE
AG-FP	0.75[d]	0.874[d]	0.611[d]	0.838[d]	0.805[d]	0.555[d]	**0.935**[a]	1.018[a]	0.622[a]
BT-FP	0.763[d]	0.855[d]	0.609[d]	0.895[d]	0.643[d]	0.332[d]	0.919[c]	1.125[c]	0.705[c]
BT_s-FP	0.829[d]	0.747[d]	0.536[d]	0.903[d]	0.621[d]	0.294[d]	0.933[c]	1.036[c]	**0.575**[c]
AGBT-FP	0.781[d]	0.824[d]	0.604[d]	0.885[d]	0.677[d]	0.404[d]	0.933[c]	**0.994**[c]	0.594[c]
$AGBT_s$-FP	**0.83**[d]	**0.743**[d]	**0.527**[d]	**0.905**[d]	**0.615**[d]	**0.299**[d]	0.931[c]	1.039[c]	0.583[c]
Datasets	Lipophilicity			BBBP					
Metric	R^2	RMSE	MAE	AUC-ROC	Accuracy				
AG-FP	0.699[a]	0.664[a]	0.492[a]	0.677[b]	0.559[b]				
BT-FP	0.726[c]	0.626[c]	0.466[c]	0.736[c]	**0.642**[c]				
BT_s-FP	0.774[c]	**0.570**[c]	**0.411**[c]	**0.763**[b]	0.632[b]				
AGBT-FP	0.711[c]	0.663[c]	0.504[c]	0.738[a]	0.623[a]				
$AGBT_s$-FP	**0.776**[a]	0.579[a]	0.418[a]	0.761[b]	0.632[b]				

* Best performances are produced on [a] GBDT, [b] RF, [c] STDNN, and [d] MTDNN;

Supplementary Table 8: Standard deviation of R^2, RMSE, and MAE on FreeSolv and Lipophilicity datasets for 10 replicate experiments. To eliminate systematic errors in the machine learning models, for each machine learning algorithm in each experiment, the consensus of the predicted values from 20 different models (generated with different random seeds) was taken for each molecule.

Datasets	FreeSolv			Lipophilicity		
Metric	R^2	RMSE	MAE	R^2	RMSE	MAE
AG-FP	±0.030	±0.275	±0.106	±0.035	±0.029	±0.018
BT-FP	±0.024	±0.291	±0.112	±0.030	±0.034	±0.020
BT_s-FP	±0.022	±0.236	±0.100	±0.019	±0.026	±0.013
AGBT-FP	±0.027	±0.217	±0.090	±0.038	±0.029	±0.020
$AGBT_s$-FP	±0.020	±0.197	±0.090	±0.024	±0.019	±0.010

References

[1] Anna Gaulton, Anne Hersey, Michał Nowotka, A Patricia Bento, Jon Chambers, David Mendez, Prudence Mutowo, Francis Atkinson, Louisa J Bellis, Elena Cibrián-Uhalte, et al. The chembl database in 2017. *Nucleic acids research*, 45(D1):D945–D954, 2017.

[2] Anna Gaulton, Louisa J Bellis, A Patricia Bento, Jon Chambers, Mark Davies, Anne Hersey, Yvonne Light, Shaun McGlinchey, David Michalovich, Bissan Al-Lazikani, et al. Chembl: a large-scale bioactivity database for drug discovery. *Nucleic acids research*, 40(D1):D1100–D1107, 2012.

[3] Kedi Wu and Guo-Wei Wei. Quantitative toxicity prediction using topology based multitask deep neural networks. *Journal of chemical information and modeling*, 58(2):520–531, 2018.

[4] Kevin S Akers, Glendon D Sinks, and T Wayne Schultz. Structure–toxicity relationships for selected halogenated aliphatic chemicals. *Environmental toxicology and pharmacology*, 7(1):33–39, 1999.

[5] Hao Zhu, Alexander Tropsha, Denis Fourches, Alexandre Varnek, Ester Papa, Paola Gramatica, Tomas Oberg, Phuong Dao, Artem Cherkasov, and Igor V Tetko. Combinatorial qsar modeling of chemical toxicants tested against tetrahymena pyriformis. *Journal of chemical information and modeling*, 48(4): 766–784, 2008.

[6] Tiejun Cheng, Yuan Zhao, Xun Li, Fu Lin, Yong Xu, Xinglong Zhang, Yan Li, Renxiao Wang, and Luhua Lai. Computation of octanol- water partition coefficients by guiding an additive model with knowledge. *Journal of chemical information and modeling*, 47(6):2140–2148, 2007.

[7] Zhenqin Wu, Bharath Ramsundar, Evan N Feinberg, Joseph Gomes, Caleb Geniesse, Aneesh S Pappu, Karl Leswing, and Vijay Pande. Moleculenet: a benchmark for molecular machine learning. *Chemical science*, 9(2):513–530, 2018.

[8] John S Delaney. Esol: estimating aqueous solubility directly from molecular structure. *Journal of chemical information and computer sciences*, 44(3):1000–1005, 2004.

[9] David L Mobley and J Peter Guthrie. Freesolv: a database of experimental and calculated hydration free energies, with input files. *Journal of computer-aided molecular design*, 28(7):711–720, 2014.

[10] M Wenlock and N Tomkinson. Experimental in vitro dmpk and physicochemical data on a set of publicly disclosed compounds. 2015.

[11] Ines Filipa Martins, Ana L Teixeira, Luis Pinheiro, and Andre O Falcao. A bayesian approach to in silico blood-brain barrier penetration modeling. *Journal of chemical information and modeling*, 52(6): 1686–1697, 2012.

[12] Jacob Devlin, Ming-Wei Chang, Kenton Lee, and Kristina Toutanova. Bert: Pre-training of deep bidirectional transformers for language understanding. *arXiv preprint arXiv:1810.04805*, 2018.

[13] Ashish Vaswani, Noam Shazeer, Niki Parmar, Jakob Uszkoreit, Llion Jones, Aidan N Gomez, Łukasz Kaiser, and Illia Polosukhin. Attention is all you need. In *Advances in neural information processing systems*, pages 5998–6008, 2017.

[14] Myle Ott, Sergey Edunov, Alexei Baevski, Angela Fan, Sam Gross, Nathan Ng, David Grangier, and Michael Auli. fairseq: A fast, extensible toolkit for sequence modeling. *arXiv preprint arXiv:1904.01038*, 2019.

[15] Adam Paszke, Sam Gross, Soumith Chintala, Gregory Chanan, Edward Yang, Zachary DeVito, Zeming Lin, Alban Desmaison, Luca Antiga, and Adam Lerer. Automatic differentiation in pytorch. 2017.

[16] Diederik P Kingma and Jimmy Ba. Adam: A method for stochastic optimization. *arXiv preprint arXiv:1412.6980*, 2014.

[17] Kristopher Opron, Kelin Xia, and Guo-Wei Wei. Communication: Capturing protein multiscale thermal fluctuations, 2015.

[18] Duc D Nguyen, Tian Xiao, Menglun Wang, and Guo-Wei Wei. Rigidity strengthening: A mechanism for protein–ligand binding. *Journal of chemical information and modeling*, 57(7):1715–1721, 2017.

[19] Kelin Xia, Kristopher Opron, and Guo-Wei Wei. Multiscale gaussian network model (mgnm) and multiscale anisotropic network model (manm). *The Journal of chemical physics*, 143(20):11B616_1, 2015.

[20] Kaifu Gao, Duc Duy Nguyen, Vishnu Sresht, Alan M Mathiowetz, Meihua Tu, and Guo-Wei Wei. Are 2d fingerprints still valuable for drug discovery? *Physical Chemistry Chemical Physics*, 22(16):8373–8390, 2020.

[21] Jian Jiang, Rui Wang, Menglun Wang, Kaifu Gao, Duc Duy Nguyen, and Guo-Wei Wei. Boosting tree-assisted multitask deep learning for small scientific datasets. *Journal of Chemical Information and Modeling*, 60(3):1235–1244, 2020.

[22] T Martin et al. User's guide for test (version 4.2)(toxicity estimation software tool): A program to estimate toxicity from molecular structure. *Washington (USA): US-EPA*, 2016.

[23] Bao Wang, Chengzhang Wang, Kedi Wu, and Guo-Wei Wei. Breaking the polar-nonpolar division in solvation free energy prediction. *Journal of computational chemistry*, 39(4):217–233, 2018.

[24] Fabian Pedregosa, Gaël Varoquaux, Alexandre Gramfort, Vincent Michel, Bertrand Thirion, Olivier Grisel, Mathieu Blondel, Peter Prettenhofer, Ron Weiss, Vincent Dubourg, et al. Scikit-learn: Machine learning in python. *the Journal of machine Learning research*, 12:2825–2830, 2011.

[25] Stephen J Capuzzi, Regina Politi, Olexandr Isayev, Sherif Farag, and Alexander Tropsha. Qsar modeling of tox21 challenge stress response and nuclear receptor signaling toxicity assays. *Frontiers in Environmental Science*, 4:3, 2016.

[26] Bharath Ramsundar, Bowen Liu, Zhenqin Wu, Andreas Verras, Matthew Tudor, Robert P Sheridan, and Vijay Pande. Is multitask deep learning practical for pharma? *Journal of chemical information and modeling*, 57(8):2068–2076, 2017.

[27] Jan Wenzel, Hans Matter, and Friedemann Schmidt. Predictive multitask deep neural network models for adme-tox properties: learning from large data sets. *Journal of chemical information and modeling*, 59(3):1253–1268, 2019.

[28] Kedi Wu, Zhixiong Zhao, Renxiao Wang, and Guo-Wei Wei. Topp–s: Persistent homology-based multi-task deep neural networks for simultaneous predictions of partition coefficient and aqueous solubility. *Journal of computational chemistry*, 39(20):1444–1454, 2018.

[29] Abdul Karim, Avinash Mishra, MA Hakim Newton, and Abdul Sattar. Efficient toxicity prediction via simple features using shallow neural networks and decision trees. *Acs Omega*, 4(1):1874–1888, 2019.

[30] Jiarui Chen, Hong-Hin Cheong, and Shirley Weng In Siu. Bestox: A convolutional neural network regression model based on binary-encoded smiles for acute oral toxicity prediction of chemical compounds. In *International Conference on Algorithms for Computational Biology*, pages 155–166. Springer, 2020.

[31] Duc Duy Nguyen and Guo-Wei Wei. Dg-gl: Differential geometry-based geometric learning of molecular datasets. *International journal for numerical methods in biomedical engineering*, 35(3):e3179, 2019.
