# Supplementary material for: Algebraic graph-assisted bidirectional transformers for molecular property prediction
Source: Nat Commun. 2021 Jun 10;12:3521. doi: 10.1038/s41467-021-23720-w (PMC8192505; doi:10.1038/s41467-021-23720-w)
Supplement: Supplementary file 3 — Description of Additional Supplementary Files [file 41467_2021_23720_MOESM3_ESM.pdf]

Title: Supplementary Data 1

Description:

- Sheet 'TableS6_Lipophilicity' contains all results for the dataset Lipophilicity in the Supplementary Table 6, including the machine learning prediction results for five fingerprints BT_s-FP, AGBT_s-FP, BT-FP, AGBT-FP, and AG-FP. For each fingerprint, we use three machine learning algorithms, gradient boosting regression tree (GBRT), single-task deep neural network (ST-DNN), and random forest (RF), to compute 10 different data splits. Three metrics, mean absolute error (MAE), root mean squared error (RMSE), and the squared Pearson correlation coefficient (pearsonr2), are used to evaluate the machine learning models. The mean and standard deviation of the prediction results for the 10 different data splits have been highlighted in the file.

- Sheet 'TableS6_FreeSolv' contains all results for the dataset FreeSolv in the Supplementary Table 6, including the machine learning prediction results for five fingerprints BT_s-FP, AGBT_s-FP, BT-FP, AGBT-FP, and AG-FP. For each fingerprint, we use three machine learning algorithms, gradient boosting regression tree (GBRT), single-task deep neural network (ST-DNN), and random forest (RF), to compute 10 different data splits. Three metrics, mean absolute error (MAE), root mean squared error (RMSE), and the squared Pearson correlation coefficient (pearsonr2), are used to evaluate the machine learning models. The mean and standard deviation of the prediction results for the 10 different data splits have been highlighted in the file.
