# Supplementary material for: Algebraic graph-assisted bidirectional transformers for molecular property prediction
Source: Nat Commun. 2021 Jun 10;12:3521. doi: 10.1038/s41467-021-23720-w (PMC8192505; doi:10.1038/s41467-021-23720-w)
Supplement: Supplementary file 4 — Reporting Summary [file 41467_2021_23720_MOESM4_ESM.pdf]

nature research

Corresponding author(s): Guowei Wei, Feng Pan

Last updated by author(s): May 1, 2021

Reporting Summary

Nature Research wishes to improve the reproducibility of the work that we publish. This form provides structure for consistency and transparency in reporting. For further information on Nature Research policies, see our Editorial Policies and the Editorial Policy Checklist.

Statistics

For all statistical analyses, confirm that the following items are present in the figure legend, table legend, main text, or Methods section.

n/a	Confirmed	
[ ]	[x]	The exact sample size (n) for each experimental group/condition, given as a discrete number and unit of measurement
[ ]	[x]	A statement on whether measurements were taken from distinct samples or whether the same sample was measured repeatedly
[x]	[ ]	The statistical test(s) used AND whether they are one- or two-sided<br>*Only common tests should be described solely by name; describe more complex techniques in the Methods section.*
[ ]	[x]	A description of all covariates tested
[ ]	[x]	A description of any assumptions or corrections, such as tests of normality and adjustment for multiple comparisons
[ ]	[x]	A full description of the statistical parameters including central tendency (e.g. means) or other basic estimates (e.g. regression coefficient) AND variation (e.g. standard deviation) or associated estimates of uncertainty (e.g. confidence intervals)
[x]	[ ]	For null hypothesis testing, the test statistic (e.g. F, t, r) with confidence intervals, effect sizes, degrees of freedom and P value noted<br>*Give P values as exact values whenever suitable.*
[x]	[ ]	For Bayesian analysis, information on the choice of priors and Markov chain Monte Carlo settings
[ ]	[x]	For hierarchical and complex designs, identification of the appropriate level for tests and full reporting of outcomes
[ ]	[x]	Estimates of effect sizes (e.g. Cohen's d, Pearson's r), indicating how they were calculated

Our web collection on statistics for biologists contains articles on many of the points above.

Software and code

Policy information about availability of computer code

Data collection	The overall codes have been released as an open-source code in the Github repository: https://github.com/ChenDdon/AGBTcode.
Data analysis	The data analysis tool used in this paper is the open source software Python and its related libraries. A statement of the required libraries is given in the 'Requirments' in the Github repository https://github.com/ChenDdon/AGBTcode..

For manuscripts utilizing custom algorithms or software that are central to the research but not yet described in published literature, software must be made available to editors and reviewers. We strongly encourage code deposition in a community repository (e.g. GitHub). See the Nature Research guidelines for submitting code & software for further information.

Data

Policy information about availability of data

All manuscripts must include a data availability statement. This statement should provide the following information, where applicable:

- Accession codes, unique identifiers, or web links for publicly available datasets
- A list of figures that have associated raw data
- A description of any restrictions on data availability

The pre-training dataset used in this work is CheMBL26, which is available at https://ftp.ebi.ac.uk/pub/databases/chembl/ChEMBLdb/releases/chembl_26/. To ensure the reproducibility of this work, the eight datasets used in this work, including four quantitative toxicity datasets (LD50, IGC50, LC50, and LC50DM), partition coefficient dataset, FreeSolv dataset, Lipophilicity dataset and BBBP dataset, are publically available at https://weilab.math.msu.edu/Database/.

Field-specific reporting

Please select the one below that is the best fit for your research. If you are not sure, read the appropriate sections before making your selection.

☐ Life sciences ☐ Behavioural & social sciences ☒ Ecological, evolutionary & environmental sciences

For a reference copy of the document with all sections, see nature.com/documents/nr-reporting-summary-flat.pdf

Ecological, evolutionary & environmental sciences study design

All studies must disclose on these points even when the disclosure is negative.

Study description

We propose an algebraic graph-assisted bidirectional transformer (AGBT) framework by fusing representations generated by algebraic graph and bidirectional transformer, as well as a variety of machine learning algorithms, including decision trees, multitask learning, and deep neural networks. We validate the proposed AGBT framework on eight molecular datasets, involving quantitative toxicity, physical chemistry, and physiology datasets. Extensive numerical experiments have shown that AGBT is a state-of-the-art framework for molecular property prediction.

Research sample

LD50 measures the number of chemicals that can kill half of the rats when orally ingested. The LD50 represents the amount of chemicals that can kill half of the rats when orally ingested. It was originally from https://chem.nlm.nih.gov/chemidplus/. IGC50 records the 50% growth inhibitory concentration of Tetrahymena pyriformis organism after 40h. LC50 reports at the concentration of test chemicals in the water in milligrams per liter that cause 50% of fathead minnows to die after 96h. The last one is LC50DM, which represents the concentration of test chemicals in the water in milligrams per liter that cause 50% Daphnia Magna to die after 48h. LC50 and LC50DM were originally from http://cfpub.epa.gov/ecotox/. The unit of toxicity reported in these four data sets is -log_10 mol/L. The sizes of these four data sets vary from 353 to 7413, which poses a challenge for a predictive model to achieve consistent accuracy and robustness. For the partition coefficient, the octanol-water partition coefficients, prediction task, the training set contained 8199 molecules and the test set included 406 components. All components in the test set were approved as organic drugs by the Food and Drug Administration (FDA). The logP values, for all training and test sets were compiled by Cheng et al., and all logP values ranged from -4.64 to 8.42.

The three datasets Free Solvation (FreeSolv), Lipophilicity, and the Blood–brain barrier penetration (BBBP) are derived from the work of MoleculeNet. ESOL contains 1128 molecules paired with aqueous solubility. This dataset has been used to estimate aqueous solubility directly from molecular structure.. The FreeSolv dataset contains 643 compounds, and the labels include both experimental and calculated hydration free energy of small molecules in water. The unit of the label is kilocalorie per mole (kcal/mol). Lipophilicity is a dataset contains 4200 compounds, which are derived from ChEMBL database. The measured octanol/water distribution coefficient (logD) of the compound was used as the label. For the task of classification, the Blood-brain barrier penetration (BBBP) dataset is used in this study. BBBP contains 2042 small molecules and original from a study on the modeling and prediction of the barrier permeability.The binary labels for compound permeability properties are used in this study.

The detailed descriptions are provided in the 'Datasets' section of Supporting Information.

Sampling strategy

In order to make a fair comparison, all data divisions follow the practice of published articles.

For LD50, IGC50, LC50, and LC50DM datasets, they were pre-split into the training and test sets in the way following the literature. For the FreeSolv and Lipophilicity datasets, which are adopted from MoleculeNet, we randomly split these datasets (following the same procedure as in MoleculeNet) 10 times. Following MoleculeNet, scaffold splitting is used to split the BBBP dataset into training, validation, and test set follows the ratio of 80/10/10.

Data collection

All the data are publically available. For the pre-training dataset used in this work is CheMBL26, which is available at https://ftp.ebi.ac.uk/pub/databases/chembl/ChEMBLdb/releases/chembl_26/. For the other eight datasets used in this work, including four quantitative toxicity datasets (LD50, IGC50, LC50, and LC50DM), partition coefficient dataset, FreeSolv dataset, Lipophilicity dataset and BBBP dataset, are publically available at https://weilab.math.msu.edu/Database/.

Timing and spatial scale

The the version of open-source data ChEMBL used in this work is ChEMBL26, which is released on website at https://ftp.ebi.ac.uk/pub/databases/chembl/ChEMBLdb/releases/chembl_26/. The eight datasets used in this work are generated from published works. And collected in the website https://weilab.math.msu.edu/Database/. The CheMBL26 contains 1936342 molecules. The LD50, IGC50, LC50, and LC50DM datasets contain 7413, 1792, 823, and 353 molecules respectively. And the partition coefficient dataset, FreeSolv dataset, Lipophilicity dataset and BBBP dataset are including 8605, 643, 4200, and 2042 molecules, respectively. The detail description of the data is shown in Supplementary Datasets.

Data exclusions

No data were excluded from the analyses. All data used in this study are following the published works.

Reproducibility

To eliminate systematic errors in the machine learning models, for each machine learning algorithm, the consensus of the predicted values from 20 different models (generated with different random seeds) was taken for each molecule. Note that the consensus value here refers to the average of the predicted results from different models for each molecule of each specific training-test splitting. All the final results are reproducible.

Randomization

For LD50, IGC50, LC50, and LC50DM datasets, they were pre-split into the training and test sets in the way following the literature. For the FreeSolv and Lipophilicity datasets, which are adopted from MoleculeNet, we randomly split these datasets (following the same procedure as in MoleculeNet) 10 times. Following MoleculeNet, scaffold splitting is used to split the BBBP dataset into training, validation, and test set follows the ratio of 80/10/10.

Blinding

We validate the proposed model on eight datasets, including four toxicity datasets, partition coefficient dataset, FreeSolv dataset, Lipophilicity dataset and BBBP dataset, which are given in the published work. For fair comparison, all data are consistent with

published work, and the corresponding data splitting are consistent. No data were discarded.

Did the study involve field work? ☐ Yes ☒ No

Reporting for specific materials, systems and methods

We require information from authors about some types of materials, experimental systems and methods used in many studies. Here, indicate whether each material, system or method listed is relevant to your study. If you are not sure if a list item applies to your research, read the appropriate section before selecting a response.

Materials & experimental systems

n/a	Involved in the study
☒	☐ Antibodies
☒	☐ Eukaryotic cell lines
☒	☐ Palaeontology and archaeology
☒	☐ Animals and other organisms
☒	☐ Human research participants
☒	☐ Clinical data
☒	☐ Dual use research of concern

Methods

n/a	Involved in the study
☒	☐ ChIP-seq
☒	☐ Flow cytometry
☒	☐ MRI-based neuroimaging
